# The Identification and Characterization of the *PeGRF* Gene Family in *Populus euphratica* Oliv. Heteromorphic Leaves Provide a Theoretical Basis for the Functional Study of *PeGRF9*

**DOI:** 10.3390/ijms26010066

**Published:** 2024-12-25

**Authors:** Ying Wang, Zhihua Wu, Mingyu Jia, Jing Li, Tongrui Song, Hongyan Jin, Jianhao Sun, Chen Qiu, Xiaona Lu, Yang Yuan, Yongqiang Chen, Peipei Jiao, Zhijun Li

**Affiliations:** 1 Xinjiang Production and Construction Corps Key Laboratory of Protection and Utilization of Biological Resources in Tarim Basin, College of Life Science, Tarim University, Alar 843300, China; 15235060710@163.com (Y.W.); jiamingyu0717@163.com (M.J.); jing926819729@163.com (J.L.); songtr20001209@163.com (T.S.); j2601803054@163.com (H.J.); sunjianhaotea@163.com (J.S.); qiuchentea@163.com (C.Q.); 2 College of Life Sciences, Zhejiang Normal University, Jinhua 321004, China; zhuawu@zjnu.edu.cn (Z.W.); xiaonalu@zjnu.edu.cn (X.L.); 3 Key Laboratory of Crop Genetic Improvement, Hubei Hongshan Laboratory, Huazhong Agricultural University, Wuhan 430070, China; yangyuan9109@163.com (Y.Y.); chenyq2017@163.com (Y.C.)

**Keywords:** *Populus euphratica*, GRF (growth-regulating factor), genetic family identification, heteromorphic leaves

## Abstract

*Populus euphratica* Oliv. typically has four kinds of heteromorphic leaves: linear (Li), lanceolate (La), ovate (Ov) and broad ovate (Bo). Heteromorphic leaves help *P. euphratica* adapt to extreme desert environments and further contribute to protection against land desertification in Northwest China. In the authors’ previous research, growth-regulating factors (GRFs) were speculated to be related to the development of *P. euphratica* heteromorphic leaves via multi-omics integrated analysis. However, the genomic features and biological role of the *P. euphratica GRF* gene family in heteromorphic leaves are still unclear. In this study, 19 *PeGRF* genes were genome-widely identified and characterized in *P. euphratica*, and their physicochemical properties, gene structure and phylogenetic evolution were analyzed. An analysis of the research showed that *PeGRFs* were unevenly distributed on 11 chromosomes and that PeGRF proteins contained conserved motif 1 (WRC) and motif 2 (QLQ). Moreover, 19, 15, 19 and 22 *GRFs* were identified in *Populus deltoides* Marshall, *Populus pruinosa* Schrenk, *Salix sinopurpurea* C. Wang et C. Y. Yang and *Salix suchowensis* W. C. Cheng, respectively. A collinearity analysis showed that the *PeGRF* family evolved slowly within Populus species. A phylogenetic tree of the GRF family was also constructed, and GRFs were divided into four subfamilies. A large number of cis-acting elements were related to plant growth and development, plant hormone response and stress response on the promoter of *PeGRFs*. The expression pattern of *PeGRFs* showed significant up-regulation in broad leaves (Ov and Bo) compared with narrow leaves (Li and La). In combination with the predicted gene regulatory network, *PeGRF9* (*PeuTF06G01147.1*) may have an important contribution to the leaf shape development of *P. euphratica.* The heterologous expression of *PeGRF9* in wild-type plants (Col-0) of *Arabidopsis thaliana* (L.) Heynh was also studied, showing a significant increase in the leaf area of overexpressing plants compared with the wild type. Nineteen *PeGRF* gene members were identified and characterized in *P. euphratica*, and a comparison of the genomic analysis of Populus GRF members revealed their evolutionary features. The further overexpression of *PeGRF9* in *A. thaliana* revealed its biological role in the heteromorphic leaves of *P. euphratica.* This study not only provides new insights into the evolution and function of PeGRFs in *P. euphratica* heteromorphic leaves but also helps in an understanding of the adaptive evolution of *P. euphratica* in drought desert environments.

## 1. Introduction

The leaf is the most sensitive organ of plants in response to environmental changes. Its morphological characteristics, such as leaf area, leaf length–width ratio, petiole length, leaf thickness and specific leaf weight, can reflect the adaptability of plants to specific environments and their survival strategies [1,2]. Therefore, leaves have often been used as research objects to reveal the adaptation and performance of plants to different environments in the process of survival and development [3]. A plant usually produces only one form of mature leaf in response to its living environment. However, some plants exhibit obvious leaf shape variation in their vertical direction, producing various forms of mature leaves to respond to complex and changeable environments—a phenomenon known as “heterophylly” [4]. Leaf shape changes are usually closely related to environmental factors [5,6,7]. For example, the leaf shape changes in the aerial and aquatic leaves of *Marsilea quadrifolia* L. [8], *Ranunculus flabellaris* Raf. [9], *Hippuris vulgaris* L. [5], *Callitriche heterophylla* Pursh [10], *Potamogeton nodosus* Poir. [11] and *Trapa bispinosa* Roxb. [12] are due to environmental changes.

*Populus euphratica*, as the only natural deciduous tree in desert areas, has evolved high stress resistance and strong environmental adaptability, which play an important role in the local ecosystem [13]. *P. euphratica* showed obvious heteromorphic leaf traits, with adult trees displaying a sequential production of linear (Li, leaf index ≥ 4), lanceolate (La, 2 ≤ leaf index < 4), ovate (Ov, 1 ≤ leaf index < 2) and broad ovate (Bo, leaf index < 1) leaves as they ascend from the lower canopies to the upper canopies, accompanied by a gradual increase in leaf width and area [14]. At present, research on the heteromorphic leaves of *P. euphratica* mostly takes leaves of different shapes in the same canopy as the research object and discusses the adaptability of different leaf shapes to the environment from the perspective of morphology, structure and physiological characteristics [15]. Relevant studies have shown that the leaves of *P. euphratica* not only have obvious differences in appearance and morphology but also have certain differences in anatomical structure and functional characteristics. For example, broad leaves (Ov and Bo) show stronger resistance to stress than narrow leaves (Li and La), and this resistance is a manifestation of its long-term adaptation to the ecological environment [16]. The morphological structure and spatial distribution of various heteromorphic leaves are related to individual development stages, and the drought resistance characteristics of the morphological and anatomical structure of heteromorphic leaves are considerably related to the development stage [17,18,19,20]. The above-mentioned results show that the differences in the heteromorphic leaves of *P. euphratica* are related to its environmental adaptability.

Growth-regulating factors (GRFs) are a class of plant-specific transcription factors first discovered in rice (*Oryza sativa*) in 2000 (*OsGRF1*) [21]. Studies have shown that the GRF gene family has been reported to contain two conserved structural domains, WRC and QLQ, in the N-terminal region of GRF proteins. The QLQ conserved domain is composed of glutamine (Gln), leucine (Leu) and glutamine (Gln), and this domain is thought to contribute to the interaction of GRF proteins with growth regulators (GRF-interacting factors, GIFs) to regulate plant growth and development [22]. Meanwhile, the WRC conserved domain is composed of tryptophan (Trp), arginine (Arg) and cysteine (Cys) [21,23]. It is a plant-specific domain that binds to DNA, and it has a nuclear localization signal and a zinc finger motif [24,25]. At present, many *GRF* family members have been identified in several plants [24,26,27,28]. GRFs are mainly involved in leaf development and abiotic stress response. The mechanism of GRFs involved in leaf development regulation has been well studied, and most GRFs promote leaf development. For example, the overexpression of *BrGRF8* in Chinese cabbage [29] and *LsaGRF5* in *Lactuca sativa* [30] yielded enhanced leaves. The overexpression of *AtGRF1* and *AtGRF2* in *A. thaliana* significantly increased leaf size [25]. *AtGRF5* and *AN3* (*ANGUSTIFOLIA3*) can jointly regulate leaf primordium cell proliferation and affect leaf shape and size [31]. *AtGRF4* plays an important role in leaf cell proliferation and cotyledon development [32]. GRFs respond to plant growth regulator induction and abiotic stress. *AtGRF7* inhibits the expression of the osmotic stress response gene *DREB2A* under non-stress conditions, thereby enhancing tolerance to salt and drought stress [33].

In the authors’ previous study, in comparison to the narrow leaves (Li and La), the broad leaves (Ov and Bo) of *P.euphratica* had a thicker cuticle, more developed palisade tissue, more layers of epidermal cells, a tighter arrangement of mesophyll cells and a larger leaf area, indicating that the structure was more aligned with a xeromorphic adaptation. [34]. On the one hand, *P. euphratica* significantly increased the photosynthetic area by increasing leaf area and forming developed palisade tissue. On the other hand, *P. euphratica* increased resistance to water diffusion and the water retention capacity by increasing the thickness of the epidermis and the cuticle, thereby reducing water loss [35]. This phenomenon is thought to be an adaptive strategy of heteromorphic leaves in *P. euphratica* to cope with a desert arid environment. However, the function of GRFs in *P. euphratica* has not been reported. Given that GRFs play an important role in the regulation of leaf growth and development and stress resistance, this study speculated that *PeGRFs* may have the same functions in leaf development in *P. euphratica*. Therefore, this study aimed to identify and analyze the members of the *PeGRF* family in combination with the previous multi-omics analysis [36]. The function of *PeGRF9* in leaf size regulation was explored by genetic engineering in *Arabidopsis*. The results could help in a comprehensive understanding of the biological characteristics of the *PeGRF* family and elucidate the role of *PeGRF9* in the development of the heteromorphic leaves of *P. euphratica*, further providing important basic data for the subsequent functional study of the *GRF* gene family of *P. euphratica*.

## 2. Results

### 2.1. Identification and Prediction of the Physicochemical Properties of PeGRF Family Members

**Figure 1:**
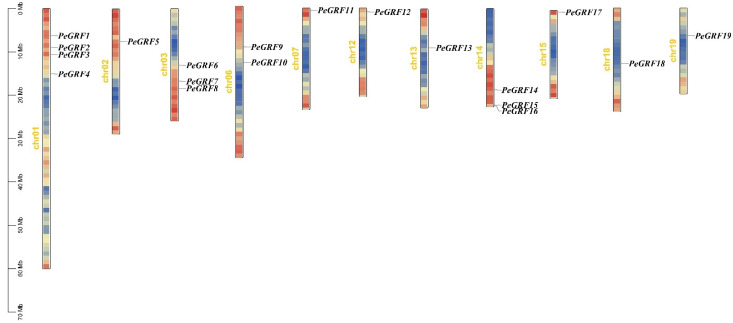
Chromosomal locations of *PeGRFs*. Blue bar indicates low gene density, and red bar indicates high gene density on chromosomes.

A total of 19 *PeGRFs* were identified in the *P. euphratica* genome, and they were named *PeGRF1*–*PeGRF19* in accordance with their location on the chromosome (Figure 1). An analysis of PeGRF protein characteristics showed that the *PeGRF8* protein had the shortest length (201 amino acids), *PeGRF15* had the longest length (609 amino acids) and the average length of 12 GRF proteins was 396.37 amino acids. The molecular weight of the GRF proteins ranged from 22.32 kD to 66.16 kD, with an average molecular weight of 43.71 kD. *PeGRF7* had the minimum molecular weight, whereas *PeGRF15* had the maximum. The minimum isoelectric point of a PeGRF protein was 6.54 (*PeGRF16*), and the maximum was 9.82 (*PeGRF2*). The average isoelectric point was 8.42. Meanwhile, 95% of the *PeGRF* gene family encoded proteins with a pI greater than 7, indicating that most proteins encoded by the *P. euphratica* GRF gene family are enriched in basic amino acids. Protein hydrophobicity ranged from −0.819 (*PeGRF4*) to −0.449 (*PeGRF19*). The greater the negative value of hydrophobicity, the higher the hydrophilicity; the greater the positive value, the higher the hydrophobicity. Amphoteric amino acids had hydrophobic values ranging from −0.5 to 0.5. Amongst the PeGRF proteins, *PeGRF19* was found to be an amphoteric amino acid, whereas the others were hydrophilic proteins. The results of subcellular localization showed that all the genes were located in the nucleus, so PeGRFs may function in the nucleus as a regulator (Table 1). Overall, PeGRFs possibly have relatively conservative biological functions.

The chromosomal localization analysis of the 19 *PeGRFs* showed that they were located on 11 different chromosomes (Figure 1). The number of *PeGRFs* on chromosome 1 was the highest, with four *PeGRF* members, followed by chromosomes 3 and 14, with three *PeGRF* members, with two *PeGRF* members on chromosome 6 and one *PeGRF* member on chromosomes 2, 7, 12, 13, 16, 18 and 19. This result showed that the PeGRF family members have an asymmetric distribution pattern on chromosomes.

### 2.2. Analysis of Gene and Protein Structure of PeGRFs

**Figure 2:**
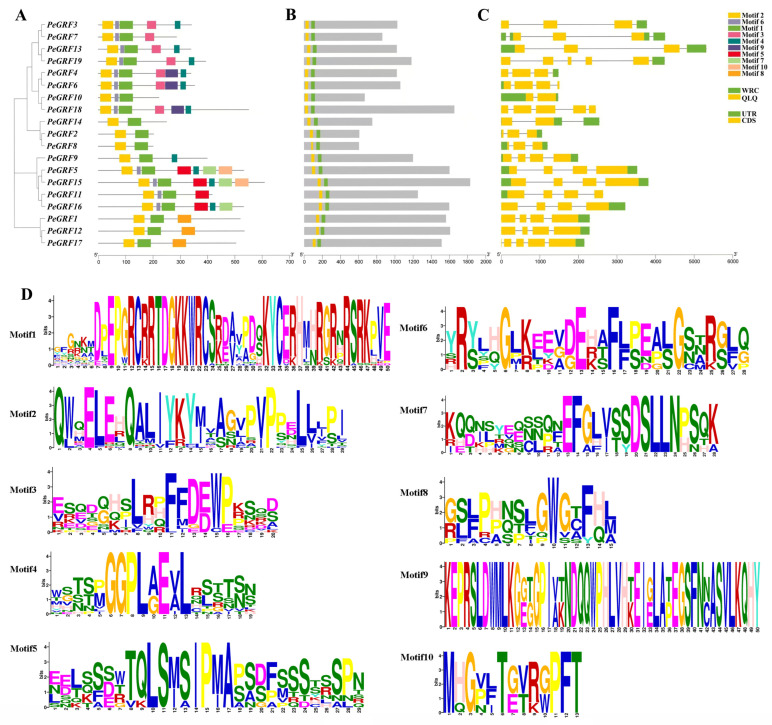
Gene structure and conserved domain of GRFs in *Populus euphratica*. (**A**) Evolutionary relationship and conserved motif. (**B**) Conserved domain—green boxes represent WRC (Trp–Arg–Cys), and yellow boxes represent QLQ (Gln–Leu–Gln). (**C**) Gene structure—yellow boxes represent exons, black lines represent introns, and green boxes represent upstream and downstream noncoding regions. (**D**) The PeGRFs have 10 conserved motifs; single-letter abbreviations represent amino acids.

The *PeGRFs* were identified by their conserved domain, conserved motif and gene structure. A motif is a typical sequence or a structure with biological functions. All members of the GRF family contain typical conserved motifs, namely, motif 1 (WRC) and motif 2 (QLQ), which are the recognition features of the family (Figure 2A,B,D). The conserved domain alignment analysis showed that the 19 PeGRFs had WRC and QLQ domains, consistent with the typical conserved motifs of the GRF family. Moreover, most of the PeGRFs had more than three motifs, indicating a function divergence in *P. euphratica.* The gene structure of each member of the *P. euphratica GRF* gene family, including intron and exon group maps, was analyzed on the basis of the positional information of the genes. The results revealed that each member had 2–5 exons and 1–5 introns (Figure 2C). Two genes, *PeGRF10* and *PeGRF14*, had two exons; *PeGRF2*, *PeGRF3*, *PeGRF4*, *PeGRF7*, *PeGRF8* and *PeGRF13* had three exons; *PeGRF1*, *PeGRF5*, *PeGRF6*, *PeGRF9*, *PeGRF11*, *PeGRF12*, *PeGRF15* and *PeGRF16* had four exons; and *PeGRF17*, *PeGRF18* and *PeGRF19* had five exons. *PeGRF10* had one intron; *PeGRF2*, *PeGRF8* and *PeGRF14* had two introns; *PeGRF1*, *PeGRF4*, *PeGRF5*, *PeGRF6*, *PeGRF9*, *PeGRF11*, *PeGRF12*, *PeGRF13*, *PeGRF15* and *PeGRF16* had three introns; *PeGRF17* and *PeGRF18* had four introns; and *PeGRF19* had five introns. In addition, seven *PeGRFs* had UTRs at both ends, whereas the remaining eight *PeGRFs* had one end of a UTR only. These results indicate that *PeGRF* family members exhibit variability and diversity in structure, likely arising from evolutionary mutations.

### 2.3. Prediction of Cis-Acting Elements in the Promoter Regions of PeGRFs

**Figure 3:**
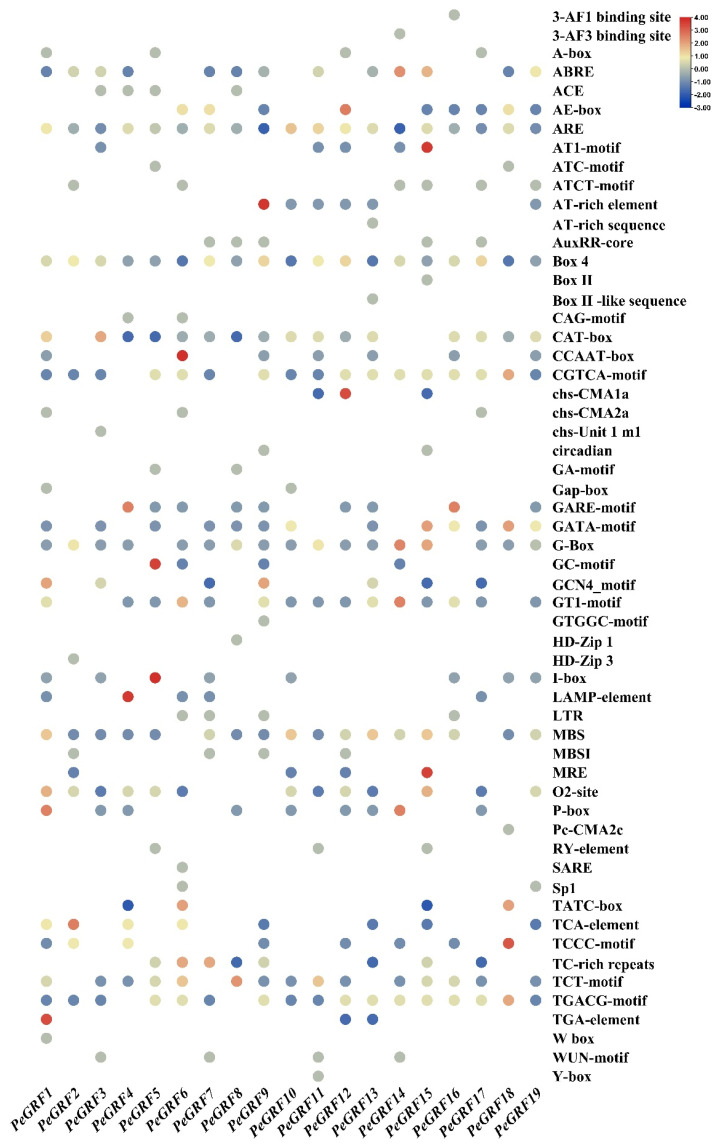
Cis-acting elements of *PeGRFs* genes displayed graphically. The number of genes is ranked from red (highest) to blue (lowest).

The upstream 2000 bp promoter region of all *PeGRFs* was predicted to further understand the function of *PeGRF*. In *P. euphratica*, the *PeGRF* gene family contains 57 elements, which are divided into plant hormone response, stress response and growth-related signals (Figure 3). For example, some elements are related to plant development, such as Box 4, O2-site, G-box, CAT-box, Gcn4-Motif, GT1-Motif and circadian-related cis-acting elements (circadian). Abscisic acid (ABRE), auxin (TGA and AUXRR elements), gibberellin (TATC box and P box), MeJA (CGTCA-motif) and salicylic acid (TCA element) were more prominent than other hormone response elements. Stress response-related elements are regulated by specific cis-acting motifs, including antioxidant response elements, TC-rich repeats, MYB binding sites and low-temperature responsiveness. The analysis showed that *PeGRFs* may be involved in various growth and development processes through hormone signal transduction or environmental stimulation.

### 2.4. Collinearity Analysis of GRFs in Multispecies

**Figure 4:**
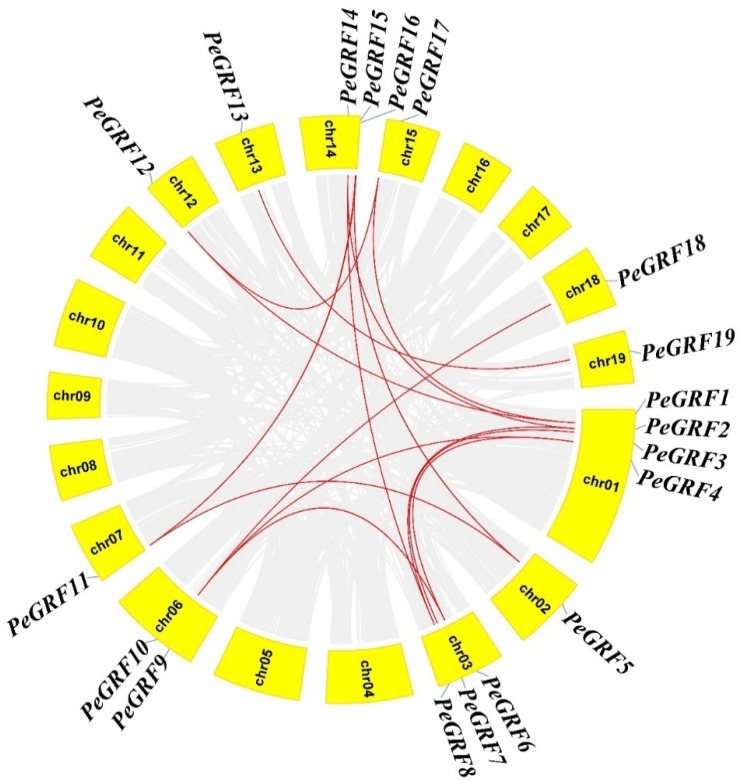
Collinearity relationship of *GRF* gene family members in *Populus euphratica*, and the red lines highlight the collinear *PeGRF* pairs.

In the process of plant evolution, the genome is expanded through gene replication. An intraspecific collinearity analysis of the *PeGRF* gene family was carried out to further explore the evolution and expansion mechanism of this family. A total of 16 gene pairs were found in the 19 *PeGRFs* of *P. euphratica* (Figure 4), implying that 16 replication events occurred. During the evolution of *PeGRFs*, the genes located on different chromosomes were differentiated, and all of them were fragmented repeat genes. The absence of gene duplication in *PeGRF9* may play an important role in the development of the *PeGRF* gene family and the *P. euphratica* genome. The Ka/Ks ratio in duplicated genes was calculated to evaluate the evolutionary rate and selection pressures (Table 2). Ka/Ks values greater than 1 generally indicate positive selection, values less than 1 indicate purification selection and values equal to 1 indicate neutral selection. The Ka/Ks calculation results for *PeGRF5*/*PeGRF11* are returning “Not a Number” (NaN), which may be due to synonymous mutations at most sites. The Ka/Ks values of the remaining 15 pairs of homologous genes were less than 1, indicating that the *PeGRF* homologous genes were purified and selected to reduce the amino acid changes caused by non-synonymous base substitutions, thereby reducing the changes in protein conformation and function. The results indicate that *PeGRF* evolves slowly. This comprehensive analysis highlights the paralogous duplication mechanisms shaping the *P. euphratica* genome.

**Figure 5:**
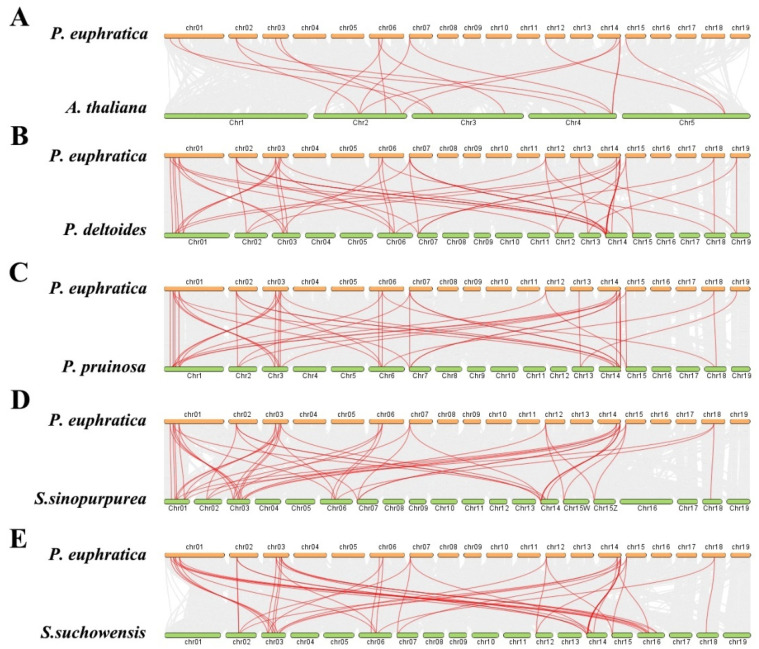
Collinearity analysis of *PeGRFs* between *P. euphratica* and five other species. (**A**) *P. euphratica* and *A. thaliana*, (**B**) *P. euphratica* and *P. deltoides*, (**C**) *P. euphratica* and *P. pruinosa*, (**D**) *P. euphratica* and *Salix sinopurpurea* and (**E**) *P. euphratica* and *Salix suchowensis*. The presence of grey lines in the background denotes collinear blocks within *P. euphratica* and other plant genomes, and the red lines emphasize collinear *GRF* pairs.

In addition to 9 GRFs in *A. thaliana*, 103 members of the *GRF* family were identified from the genomes of *P. pruinosa* (15), *P. deltoides* (19), *S. sinopurpurea* (19) and *S. suchowensis* (22) to explore the potential evolutionary process of PeGRFs in *P. euphratica*. An interspecies collinearity analysis was performed to understand the evolution and expansion mechanism of the *GRF* gene family in the genomes of *P. euphratica* and other species. The results showed that 17, 49, 51, 47 and 46 *GRF* collinear pairs were identified between *P. euphratica* and *A. thaliana*, *P. pruinosa*, *P. deltoides*, *S. sinopurpurea* and *S. suchowensis* (Figure 5), revealing that the collinearity of *GRFs* within Populus species was more conservative than that within non-Populus species. The collinearity analysis of *GRFs* in Salicaceae plants showed that the *GRF* gene family was relatively conservative, without rapid expansion and loss. Notably, 193 pairs of *PeGRFs* showed collinearity between *P. euphratica* and the other four Salicaceae plants, indicating that the collinearity fragments contained in these *GRFs* may be older than the differentiation of ancestors. The retention of *GRFs* includes their collinearity fragments, which may be caused by genome-wide replication in Salicaceae [37,38].

### 2.5. Phylogenetic Tree of PeGRFs

**Figure 6:**
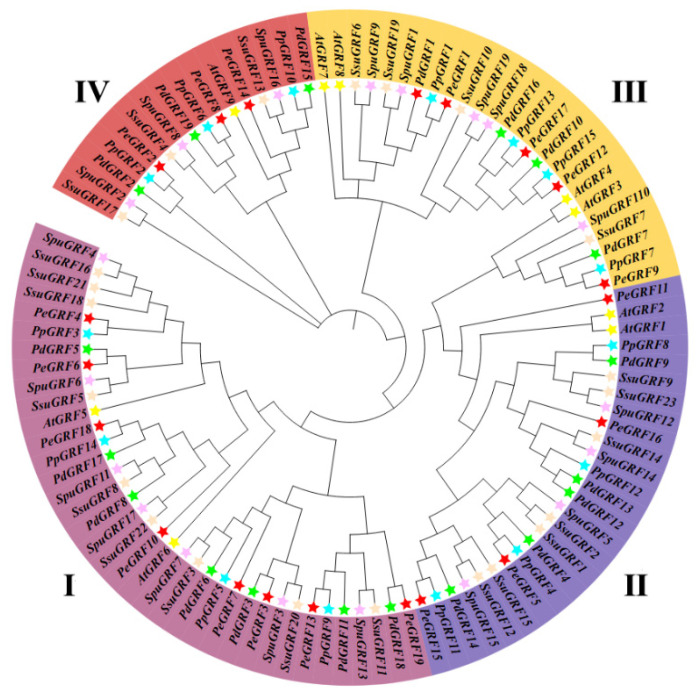
Neighbor-joining (NJ) tree of GRF gene family members in six species. Differently colored arcs indicate various subgroups. Five-pointed stars of different colors represent different species; according to the phylogenetic tree topology, the GRFs were divided into groups I–IV. The full names of species are as follows: Pe = *Populus euphratica*; At = *Arabidopsis thaliana*; Pp = *Populus pruinose*; Pd = *Populus deltoides*; Spu = *Salix sinopurpurea*; Ssu = *Salix suchowensis*.

The protein sequences of the 19 GRF gene family members of *P. euphratica* and the GRF gene family members of other species were used to construct a phylogenetic tree by the neighbor-joining (NJ) method. On the basis of the grouping method of the *A. thaliana* GRF family, 103 members of six species were divided into four groups (I–IV, Figure 6). The GRF families of the four selected Populus plants have highly conserved structures. Amongst them, *P. pruinosa* and *P. euphratica* have high homology. Considering that AtGRFs with similar functions usually cluster into the same subgroup, PeGRFs clustered with the same AtGRFs may have similar functions. These results are helpful in a further understanding of the biological functions of PeGRF gene family members in the growth and development of *P. euphratica*.

### 2.6. Analysis of GRF Member Expression in Four Kinds of Heteromorphic Leaves of P. euphratica

**Figure 7:**
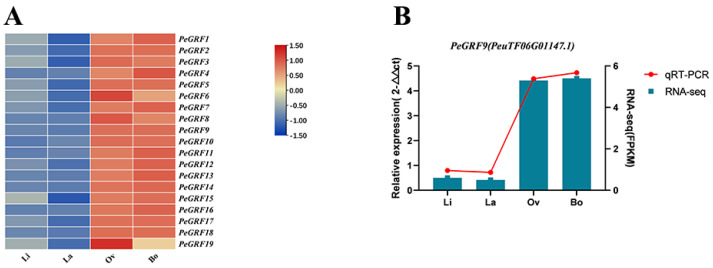
*GRF* expression patterns in heteromorphic leaves. The linear, lanceolate, ovate and broad ovate leaves are abbreviated as Li, La, Ov and Bo. (**A**) Expression patterns of *PeGRFs* during leaf development. (**B**) qRT-PCR data on the expression patterns of *PeGRF9* for heteromorphic leaves.

The expression pattern of *PeGRFs* in different leaf shapes at different leaf ages was analyzed with transcriptome data by using TBtools (version 1.119) to explore the response of *PeGRFs* to the development of the heteromorphic leaves of *P. euphratica*. The results showed that most of the *GRF* gene family members were highly expressed in Ov and Bo leaves (Figure 7A). Amongst them, one gene, *PeGRF9*, was identified by the authors’ previous multi-omics joint analysis and molecular regulatory network analysis of *P. euphratica*. The expression of *PeGRF9* was further verified by quantitative reverse transcription–polymerase chain reaction (qRT-PCR). The experimental results showed the expression of *PeGRF9* in broad leaves (Ov and Bo), which was consistent with the results of RNA-seq data (Figure 7B). In combination with the previous report of *GRFs* being involved in leaf morphogenesis and of the overexpression of *GRFs* increasing leaf area, the results suggest that *PeGRF* is involved in the development of *P. euphratica* leaf shape through different expression levels.

### 2.7. Subcellular Localization Analysis of PeGRF9

**Figure 8:**
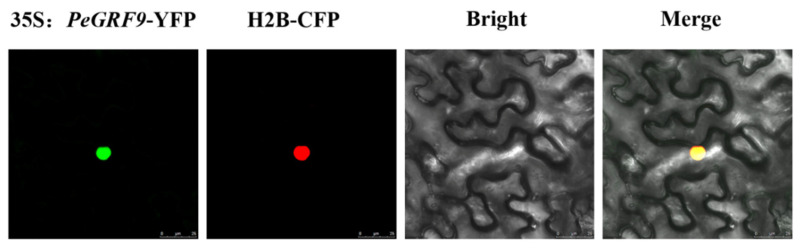
Nuclear localization of 35S:*PeGRF9*-YFP protein in tobacco leaf epidermal cells: fluorescent images of *PeGRF9* (35S:*PeGRF9*-YFP), nuclear localization signal (H2B-CFP) and merged images (35S:*PeGRF9*-YFP/H2B-CFP). Bar = 25 μm.

Through the use of the transient transformation technology of tobacco, the tobacco leaf cells were observed by laser confocal microscopy under an excitation light of 488 nm and a receiving light of 510 nm. The results showed obvious fluorescence in the nucleus of tobacco leaves, indicating that *PeGRF9* functions as a transcription factor in the nucleus (Figure 8).

### 2.8. Functional Analysis of PeGRF9 in A. thaliana

**Figure 9:**
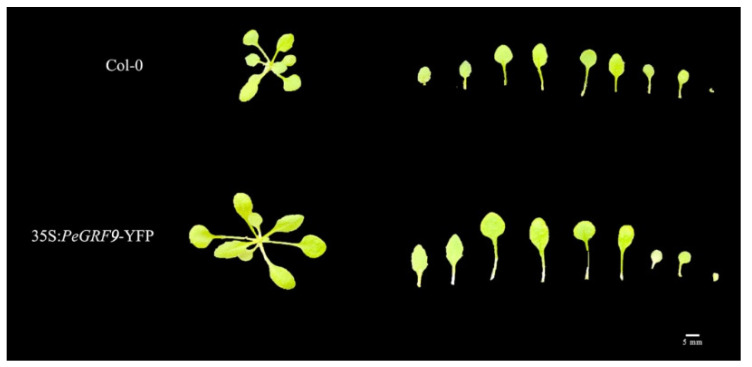
*PeGRF9* regulation of leaf morphology. The overall morphological characteristics of various plant species and the morphology of rosette leaves in different plant lines are displayed. Bar = 5 mm.

**Figure 10:**
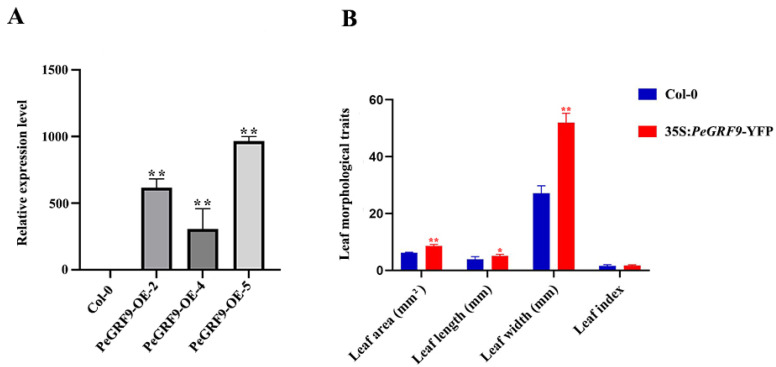
Effects of *PeGRF9* on leaf morphology changes. (**A**) Expression levels of overexpressed lines. (**B**) Fully expanded first rosette leaves of 10-day-old plants were selected for measurement. Statistically significant differences were observed compared with the wild type (* *p* < 0.05, ** *p* < 0.01).

The expression of *PeGRF9* CDS driven by the 35 S promoter in the Columbia type (Col-0) was analyzed to further explore its function in leaf shape regulation. Ten-day-old overexpressed lines and wild-type lines were photographed. The leaf area, leaf length and leaf width of the fully expanded first rosette leaves were measured, and the leaf shape index was calculated by leaf length/leaf width. The results showed that the leaves of the overexpressed transgenic plants were significantly larger than those of the wild-type plants (Figure 9). The statistical results showed that the leaf area, leaf length and leaf width of the overexpressed lines were significantly larger than those of the wild-type lines (Figure 10), but no significant difference was found in the leaf shape index. The results showed that the overexpression of *PeGRF9* regulated leaf development by promoting an increase in leaf area, leaf length and leaf width. This increase in leaf area is consistent with the larger leaf area of Ov and Bo leaves in *P. euphratica*. This finding is closely related to its more stress-resistant function, and it needs further experimental verification.

## 3. Discussion

*P. euphratica* has evolved its unique drought resistance system—heterophylly—to effectively cope with a desert arid environment and make full use of environmental resources. The structural characteristics of heteromorphic leaves make *P. euphratica* effectively maintain water, improve photosynthesis, reduce transpiration and possess strengthened tolerance to drought stress. Therefore, the heteromorphic leaves of *P. euphratica* were used as research materials to lay a foundation for an exploration of the molecular mechanism of leaf shape development of *P. euphratica* and its adaptive evolution in response to the environment.

### 3.1. Identification of GRF Family Members in Salicaceae

The GRF family is a particularly important class of growth regulators in plant leaf development. A GRF is a transcription factor unique to plants, and it plays an important role in various biological processes such as plant growth and development, hormonal response and stress response [39]. At present, 9, 12, 14, 13, 8, 20, 9, 10 and 7 *GRF* family members have been found in *Arabidopsis* [25], *Oryza* L. [23], *Zea mays* L. [40], *Solanum lycopersicum* L. [41], *Vitis vinifera* L. [42], *Musa nana* Lour. [43], *Dimocarpus longan* Lour. [44], *Prunus persica* (L.) Batsch [45] and *Cucumis melo* L. [46], respectively. However, the *GRFs* of *P. euphratica* have not been identified, and its regulatory mechanism has not been reported. Here, 19 *PeGRFs* were identified in the whole genome of *P. euphratica* by bioinformatics methods. This number is similar to the number of *GRFs* identified in the genomes of *P. pruinosa* (15), *P. deltoides* (19), *S. sinopurpurea* (19) and *S. suchowensis* (22), indicating a close relationship between Salicaceae plants in terms of evolution. A further intraspecific collinearity analysis supported this view.

### 3.2. Structural and Functional Diversity of PeGRFs

The diversity of GRFs in the C-terminal domain in plants may indicate the diversity of their biological functions [47]. Overexpression and gene knockout experiments in *A. thaliana* proved that *AtGRFs* are necessary for regulating the size of *A. thaliana* leaves and cotyledons [25]. The 14 *TaGRFs* in *Triticum aestivum* L. and all the *CsGRFs* in *Cucumis sativus* L. respond to drought and salt stress [27,48]. These results indicate that GRFs play a role in plant organ development and stress response and possess positive significance in enhancing plant resistance.

In this study, the prediction and analysis of the gene and protein structure of *PeGRF* family members showed that the *PeGRF* family members have different expression regulation patterns, indicating that *P. euphratica GRFs* are diverse. In the long process of adaptive evolution, the *GRF* gene family of *P. euphratica* has mainly been driven by purification selection, which makes the gene have functional redundancy. However, gene replication can induce functional differentiation between genes and accelerate the emergence of new genes [49]. It plays a vital role in shaping the structure and evolution of species genomes, thereby contributing to genetic adaptation and diversity among different species [50]. The results of chromosome mapping and intraspecific collinearity analysis showed that the large-scale fragment replication of the *PeGRF* gene family may lead to different roles in the development of *P. euphratica*.

The cis-element analysis of the promoter region showed that *PeGRFs* have the most growth and development-related elements, and BOX-4 existed in each family member, indicating that *PeGRFs* are closely related to plant growth and development. In addition, each *PeGRF* family member contains the abiotic stress response element ARE. A notable detail is that *PeGRF9* contains more growth and development-related elements, such as circadian elements, GCN4-motif and GT1-Motif, than other family members. Therefore, the *PeGRF* gene family is speculated to play an important role in the growth and development of *P. euphratica* and in stress. The different types and numbers of cis-acting elements in *PeGRFs* indicate the function differentiation amongst *PeGRFs*, which ensures the survival of *P. euphratica* in a harsh drought environment.

### 3.3. Functional Characterization of PeGRF9

The expression levels of *PeGRF* family members were lower in narrow leaves (Li and La) and higher in broad leaves (Ov and Bo). It is speculated that PeGRFs play a regulatory role in the development of the heteromorphic leaves of *P. euphratica*. The higher the bootstrap value of a phylogenetic tree, the higher the credibility [51]. In the phylogenetic tree, *PeGRF9* and *AtGRF4* are clustered together and have high reliability. Studies have found that *AtGRF4* is involved in regulating leaf shape [32], so *PeGRF9* may also have similar functions.

A previous multi-omics analysis found that *PeGRF9* played a key role in the regulation of the heteromorphic leaves of *P. euphratica* [36], and the transcriptome data were consistent with the results of qRT-PCR; that is, its expression in narrow leaves was lower than that in broad leaves. The leaf shape index of transgenic plants overexpressing *PeGRF9* was measured to confirm the potential function of *PeGRF9* in regulating the leaf shape development of *P. euphratica*. The leaf area of overexpressed *PeGRF9* plants increased significantly, which was consistent with the phenotype; that is, the broad leaf area of *P. euphratica* was larger than the narrow leaf area. Therefore, *PeGRF9* may promote an increase in leaf area. Heteromorphic leaves are directly related to the adaptability of *P. euphratica* to an arid desert environment. This paper focuses on the role of *PeGRF9* in regulating plant leaves, but it does not address the role of *PeGRF9* in stress resistance and other aspects, which could be further studied comprehensively.

## 4. Materials and Methods

### 4.1. Identification of GRFs

The *PeGRFs* were analyzed on the basis of *P. euphratica* genome data [52]. The hidden Markov models of the QLQ (PF08880) and WRC (PF08879) domains were obtained from the Pfam database (http://pfam.xfam.org/) and scanned to identify GRF proteins. HMMER (version 3.3.2, http://www.hmmer.org/) was used to search for proteins with a GRF domain (E < 0.001) in the entire protein sequence of *P. euphratica*. The obtained protein sequence was utilized to construct a new HMM model, and the constructed HMM model was applied to conduct a second search of all protein sequences (E < 0.001). The proteins of the *A. thaliana GRF* gene family (AtGRFs) were downloaded from Tair (https://www.arabidopsis.org/), and blastp (2.10.1+) alignment was performed with the *P. euphratica* protein sequence (E < 1 × 10^−5^).

*GRFs* from four other Salicaceae species were identified on the basis of the genome data of *P. pruinosa* (National Center for Biotechnology Information, with BioProject accession number PRJNA863418) [53], *P. deltoides* (WV94_445) [54], *S. sinopurpurea* [55] and *S. suchowensis* [22].

The ExPASy ProtParam database (https://web.expasy.org/protparam/, accessed on 16 October 2023) was used to analyze the physicochemical properties of the PeGRF genes/proteins, including coding region length, the number of amino acids (aa), molecular weight (MW) and the theoretical isoelectric point (pI). The WOLF PSORT online website (https://www.genscript.com/ accessed on 17 October 2023) was used to predict the subcellular localization of *GRF* gene family members in *P. euphratica.*

The chromosomal location of *GRFs* was obtained from the genome annotation files, and the chromosomal physical location of the *GRF* genes was displayed using TBtools.

### 4.2. Analysis of Gene Structure and Conserved Motifs of GRFs

The conserved motifs of *GRF* gene family proteins were analyzed using the MEME website (https://meme-suite.org/meme/tools/meme, accessed on 1 December 2023), and the maximum search value of the conserved motifs was set to 10.

### 4.3. Multispecies Collinearity and Phylogenetic Tree Analysis of GRFs

The protein sequence clustering and phylogenetic analysis of the GRF gene family members of *P. euphratica*, *P. plurinose*, *P. deltoides*, *S. sinopurpurea*, *S. suchowensis* and *A. thaliana* were performed using MEGA11 software (version 11.0.13). The iTOL (https://itol.embl.de/, accessed on 8 November 2023) online tool was used to edit and beautify the phylogenetic tree of the system [56]. BLASTP alignment was used to identify orthologous pairs between *P. euphratica* and five other species (*P. pruinosa*, *P. deltoides*, *S. sinopurpurea*, *S. suchowensis* and *A. thaliana*). The MCscaX function in TBtools was used to identify the collinear blocks between *P. euphratica* and other species (*P. pruinosa*, *P. deltoides*, *S. sinopurpurea*, *S. suchowensis* and *A. thaliana*).

### 4.4. Promoter Cis-Element Analysis of PeGRFs

TBtools was used to obtain the promoters of *PeGRFs* (2000 bp upstream of the translation start site). Subsequently, PlantCARE (http://bioinformatics.psb.ugent.be/webtools/plantcare/html/, accessed on 1 November 2023) was used to search for and locate cis-elements in each promoter.

### 4.5. RNA-Seq for Heteromorphic Leaves

The leaves of *P. euphratica* were collected from a *P. euphratica* forest in Alar City, Xinjiang Uygur Autonomous Region, China (latitude: 40°32′36.90″ N, longitude: 81°17′56.52″ E). From the end of July to August, the Li, La, Ov and Bo leaves of *P. euphratica* were selected, and the third or fourth sections from the base of the current-year branches were collected as experimental materials. After sampling, they were stored in a refrigerator at −80 °C, and the number of biological replicates was set at three for transcriptome sequencing. The dataset has been made available to the public for access [36] and preservation through the National Genomics Data Center (https://ngdc.cncb.ac.cn/), under project number PRJCA005959.

### 4.6. Transcriptome Sequencing and Data Analysis of PeGRFs

Whole-transcriptome sequencing by mRNA-seq was performed on an Illumina Hiseq X-Ten in Novogene (Wuhan, China) following the vendor’s recommended protocol. The clean reads were aligned against the reference genome by using HISAT2 (version 2.0.4) to estimate the expressed abundance of the annotated *P. euphratica* genes [57]. Gene expression was quantified with FPKM by using StringTie (version 2.2.1) [58].

### 4.7. Validation of PeGRF9 via qRT-PCR

The heteromorphic foliage was collected from different canopies and stored in an ultralow-temperature refrigerator at −80 °C after being rapidly frozen with liquid nitrogen. The procedure followed the methodology described in a previous publication [59]. An actin gene was used as the endogenous control. Each reaction was performed in biological triplicates, and the CT values obtained through qRT-PCR were analyzed using the 2^−ΔΔCT^ method to calculate the relative fold change values. Related primers are shown in Supplementary Table S1.

### 4.8. Experimental Material Information

The wild-type *A. thaliana* seeds used in this experiment were Columbia type (Col-0), the *Escherichia coli* competent strain was Top10, and the Agrobacterium strain was GV3101. They were all preserved in the laboratory. The overexpression vector was pGREENII 0179 (35S NOS)-YFP2. The leaf shape index of transgenic plants overexpressing *PeGRF9* was measured using T3 generation.

### 4.9. Cloning of PeGRF9 and Construction of Transgenic Plants

RNA was extracted from the leaves of *P. euphratica* by using TRIzol (Invitrogen, Waltham, MA, USA) and the cDNA RT kit with gDNA remover (Mei5 Biotechnology, Co., Ltd., Beijing, China), and was synthesized by M5Sprint qPCR. The full-length coding region of *PeGRF9* without a stop codon was amplified from cDNA or a plasmid to ensure high fidelity by using Phanta Max Super-Fidelity DNA polymerase (Vazyme, Inc., Nanjing, China). Then, it was ligated with pGREENII 0179. The *A. thaliana* plants were transformed by a flower dipping method [60,61,62]. The overexpression of *PeGRF9* was established in the wild-type background. Three lines of transgenic plants were selected for analysis.

### 4.10. Subcellular Localization of Protein Encoded by PeGRF9

*Nicotiana benthamiana* with 4-week-old leaves in good condition was selected. Agrobacterium containing *PeGRF9* and the auxiliary strain 19K that can enhance the fluorescence signal were added to an LB liquid medium for overnight culture. When the agrobacterium solution containing *PeGRF9* had an OD_600_ = 1–1.2, it was centrifuged at 10,000 rpm for 1 or 2 min. The supernatant was discarded to collect the bacteria. The configured MES solution was added to MgCl_2_·6H_2_O and acetosyringone, and KOH was used to adjust the pH to 6.0. After the above-mentioned solution was mixed with bacteria, it was allowed to stand at 28 °C for 1 h, and then the bacterial solution was injected into the back of the tobacco leaf and marked. After 24 h of dark treatment, tobacco plants were cultured in a constant-temperature culture room for 2 or 3 days. The fluorescence signal was observed by laser confocal microscopy. The microscope parameters were set as follows: an excitation light wavelength of 488 nm and a receiving light wavelength of 500–550 nm.

## 5. Conclusions

In this study, a total of 19 members of the *PeGRF* gene family were identified. Their structures, phylogenetic relationships, collinearity and cis-acting elements were analyzed. The PeGRF gene family may be involved in the formation of different leaf morphologies of *P. euphratica*. This gene family may also play an important role in the stress-related growth of *P. euphratica*. The important role of *PeGRF9* in the leaf development of *P.uphratica* was verified by RNA-seq data and qRT-PCR. The overexpression of *PeGRF9* in wild-type *A. thaliana* resulted in an increase in leaf area, which aligned with its high expression in the broad leaves and low expression in the narrow leaves of *P. euphratica*. Therefore, *PeGRF9* is speculated to promote an increase in the leaf area of *P. euphratica*. The results of this study not only provide a theoretical basis for the functional study of the PeGRF gene family in the process of leaf growth and development and stress in *P. euphratica*, but also further reveal the role of GRF genes in regulating the development of heteromorphic leaves in *P. euphratica* at the molecular level through the functional verification of *PeGRF9*.

## Figures and Tables

**Table 1:** Basic physical and chemical characteristics of the *PeGRF* gene family.

Name	Gene ID	Chromosome Location	Number of Amino Acids	Molecular Weight	Theoretical pI	Grand Average of Hydropathicity	Subcellular Localization
*PeGRF1*	PeuTF01G00792.1	Chr1: 6270156–6272451	520	56,663.69	7.21	−0.651	Nucleus
*PeGRF2*	PeuTF01G01120.1	Chr1: 8990995–8992055	202	22,524.83	9.82	−0.599	Nucleus
*PeGRF3*	PeuTF01G01318.1	Chr1: 10645697–10649477	342	38,247.66	8.7	−0.763	Nucleus
*PeGRF4*	PeuTF01G01743.1	Chr1: 15078588–15080075	340	38,212.35	8.21	−0.819	Nucleus
*PeGRF5*	PeuTF02G00947.1	Chr2: 7494925–7498451	533	58,199.61	8.61	−0.55	Nucleus
*PeGRF6*	PeuTF03G00542.1	Chr3: 13074257–13075767	353	39,523.94	8.38	−0.718	Nucleus
*PeGRF7*	PeuTF03G00943.1	Chr3: 16767556–16771807	287	31,572.14	9.23	−0.74	Nucleus
*PeGRF8*	PeuTF03G01150.1	Chr3: 18395482–18396684	201	22,327.63	9.84	−0.494	Nucleus
*PeGRF9*	PeuTF06G01147.1	Chr6: 9403104–9405098	399	43,924.25	7.27	−0.603	Nucleus
*PeGRF10*	PeuTF06G01423.1	Chr6: 12965169–12966649	222	24,928.2	9.65	−0.708	Nucleus
*PeGRF11*	PeuTF07G00075.1	Chr7: 488487–491130	417	45,472.79	9.3	−0.614	Nucleus
*PeGRF12*	PeuTF12G00087.1	Chr12: 879669–881959	535	58,299.71	8.84	−0.683	Nucleus
*PeGRF13*	PeuTF13G00777.1	Chr13: 8941639–8946959	340	37,400.2	8.22	−0.759	Nucleus
*PeGRF14*	PeuTF14G01274.1	Chr14: 18763783–18766327	250	27,699.01	8.52	−0.683	Nucleus
*PeGRF15*	PeuTF14G01745.1	Chr14: 22245773–22249589	609	66,162.14	8.42	−0.596	Nucleus
*PeGRF16*	PeuTF14G01776.1	Chr14: 22488278–22491494	532	58,424.86	6.54	−0.654	Nucleus
*PeGRF17*	PeuTF15G00068.1	Chr15: 389099–391253	504	55,041.88	6.92	−0.644	Nucleus
*PeGRF18*	PeuTF18G00553.1	Chr18: 12772180–12774632	551	62,428.96	7.41	−0.661	Nucleus
*PeGRF19*	PeuTF19G00381.1	Chr19: 6384607–6388846	394	43,475.96	8.87	−0.449	Nucleus

**Table 2:** Evolutionary selection pressures of *GRFs* in *Populus euphratica*.

Gene 1	Gene 2	Ka	Ks	Ka/Ks
*PeGRF2*	*PeGRF8*	0.11899	0.260122	0.457441
*PeGRF3*	*PeGRF7*	0.075284	0.225588	0.333725
*PeGRF4*	*PeGRF6*	0.099605	0.266788	0.373349
*PeGRF4*	*PeGRF10*	0.27853	1.85247	0.150356
*PeGRF1*	*PeGRF12*	0.591237	1.995614	0.296268
*PeGRF2*	*PeGRF14*	0.411281	1.87593	0.219241
*PeGRF1*	*PeGRF17*	0.596429	2.382612	0.250326
*PeGRF5*	*PeGRF11*	0.278972	NaN	NaN
*PeGRF5*	*PeGRF15*	0.084073	0.342507	0.245464
*PeGRF6*	*PeGRF10*	0.326551	1.203352	0.271367
*PeGRF8*	*PeGRF14*	0.502743	3.516391	0.142971
*PeGRF10*	*PeGRF18*	0.068205	0.189189	0.360513
*PeGRF11*	*PeGRF16*	0.083547	0.19539	0.427592
*PeGRF11*	*PeGRF15*	0.278326	1.619047	0.171907
*PeGRF12*	*PeGRF17*	0.136212	0.271054	0.502528
*PeGRF13*	*PeGRF19*	0.132381	0.318797	0.415252

Non-synonymous is abbreviated as Ka and synonymous is abbreviated as Ks.

## Data Availability

The RNA-seq data for *P. euphratica*’s heteromorphic leaves used in this study have been submitted to the National Genomics Data Center (https://ngdc.cncb.ac.cn/) under BioProject accession number PRJCA005959.

## Supplementary Materials

The following supporting information can be downloaded at https://www.mdpi.com/article/10.3390/ijms26010066/s1.
