# Supplementary material for: The Identification and Characterization of the PeGRF Gene Family in Populus euphratica Oliv. Heteromorphic Leaves Provide a Theoretical Basis for the Functional Study of PeGRF9
Source: Int J Mol Sci. 2024 Dec 25;26(1):66. doi: 10.3390/ijms26010066 (PMC11720670; doi:10.3390/ijms26010066)
Supplement: Supplementary file 1 [file ijms-26-00066-s001.zip › ijms-3349706-supplementary.pdf]

Supplementary Table S1. Primers used in this study

Primer Name	Primer sequence（5' to 3'）
PeGRF9-F	ACACGATCGATAAGCTTCCCATGGACTTCCATCTGAAGCAATGGAGAAAC
PeGRF9-R	TCGCCCTTGCTCACCATCCCGAAACAGTGGGGCGAGACAACTGGTG
PeGRF9-qF	TGTTAGCCGGTGCAGCTATT
PeGRF9-qR	GCTCTTCCCCAATACCCTGT
PeActin-qF	GTCCTCTTCCAGCCATCTC
PeActin-qR	TTCGGTCAGCAATACCAGG
ATUBQ10-qF	CGGAAAGCAGTTGGAGGATGG
ATUBQ10-qR	CGGAGCCTGAGAACAAGATGAAG
